# Acute Low Dose of Trazodone Recovers Glutamate Release Efficiency and mGlu2/3 Autoreceptor Impairments in the Spinal Cord of Rats Suffering From Chronic Sciatic Ligation

**DOI:** 10.3389/fphar.2020.01108

**Published:** 2020-07-17

**Authors:** Francesca Cisani, Alessandra Roggeri, Guendalina Olivero, Beatrice Garrone, Serena Tongiani, Francesco Paolo Di Giorgio, Anna Pittaluga

**Affiliations:** ^1^ Department of Pharmacy, DIFAR, Pharmacology and Toxicology Section and Center of Excellence for Biomedical Research, University of Genoa, Genoa, Italy; ^2^ Angelini RR&D (Research, Regulatory & Development), Angelini Pharma S.p.A., Rome, Italy; ^3^ IRCCS Ospedale Policlinico San Martino, Genova, Italy

**Keywords:** synaptosomes, mGlu2/3 receptor, 5-HT_2A_ receptor, glutamate exocytosis, trazodone, spinal cord, chronic sciatic ligation, neuropathic pain

## Abstract

We investigated whether chronic sciatic ligation modifies the glutamate release in spinal cord nerve endings (synaptosomes) as well as the expression and the function of presynaptic release-regulating mGlu2/3 autoreceptors and 5-HT_2A_ heteroreceptors in these particles. Synaptosomes were from the spinal cord of animals suffering from the sciatic ligation that developed on day 6 post-surgery a significant decrease of the force inducing paw-withdrawal in the lesioned paw. The exocytosis of glutamate (quantified as release of preloaded [^3^H]D-aspartate, [^3^H]D-Asp) elicited by a mild depolarizing stimulus (15 mM KCl) was significantly increased in synaptosomes from injured rats when compared to controls (uninjured rats). The mGlu2/3 agonist LY379268 (1000 pM) significantly inhibited the 15 mM KCl-evoked [^3^H]D-Asp overflow from control synaptosomes, but not in terminals isolated from injured animals. Differently, a low concentration (10 nM) of (±) DOI, unable to modify the 15 mM KCl-evoked [^3^H]D-Asp overflow in control spinal cord synaptosomes, significantly reduced the glutamate exocytosis in nerve endings isolated from the injured rats. Acute oral trazodone (TZD, 0.3 mg/kg on day 7 post-surgery) efficiently recovered glutamate exocytosis as well as the efficiency of LY379268 in inhibiting this event in spinal cord synaptosomes from injured animals. The sciatic ligation significantly reduced the expression of mGlu2/3, but not of 5-HT_2A_, receptor proteins in spinal cord synaptosomal lysates. Acute TZD recovered this parameter. Our results support the use of 5-HT_2A_ antagonists for restoring altered spinal cord glutamate plasticity in rats suffering from sciatic ligation.

## Introduction

Metabotropic glutamate (mGlu) receptors are fine tuners of the chemical transmission in the central nervous system (CNS) and represent the target of drugs proposed for the cure of neurological disorders. Naïve mGlu receptors exist as homo-dimers, as intra- (i.e. the mGlu1/mGlu5 and the mGlu2/3 receptor dimers, Longordo et al., 2006; Di Prisco et al., 2016) or inter-group heteromeric complexes (the mGlu2/mGlu4 heterodimers, Doumazane et al., 2010, the mGlu5/mGlu3 complex, Di Menna et al., 2018), or oligomerize with non-glutamatergic G protein coupled receptors to form inter-family heteromeric assemblies. It is the case of the 5-HT_2A_/mGlu2/3 receptor-receptor complex in the cortex and in the spinal cord of mammals, of the mGlu1/GABA_B_ receptor-receptor interaction in GABAergic and glutamatergic cortical terminals and of the mGlu5/A2A receptor-receptor association in the striatum (Díaz-Cabiale et al., 2002; Moreno et al., 2011; Moreno et al., 2012; Delille et al., 2012; Vergassola et al., 2018).

The allosteric properties of the interaction linking different receptors expressed within the same cells were first proposed by Agnati and colleagues in 1980 and nowadays represents a major topic to define the physio-pathological events controlling chemical transmission in CNS.

Homo- or hetero-oligomerization implies the colocalization and the physical association of receptors (i.e. the mGlu2/mGlu4 complex, Doumazane et al., 2010; mGlu2/3 – 5-HT_2A_, Moreno et al., 2012; Delille et al., 2013; Olivero et al., 2018) and functional outcomes depend on i) the reciprocal role of the receptors involved, ii) the transducing pathways they associate to, and iii) the endogenous transmitters acting at each component of the receptor complex. The intimate association of two receptor proteins to form heterodimers also implies that drugs acting at one receptor affect stereo-chemically the coupled receptor and could alter its insertion in plasmamembranes.

As far as the mGlu2/3 and the 5-HT_2A_ heterodimers are concerned, this receptor complex was first shown to exist in the cortex of mammals. Here, these receptors were reported to interact in an antagonist-like fashion, since the blockade of one receptor reinforced the signalling elicited by the other one (Moreno et al., 2011; Delille et al., 2012). This observation led to propose that the antipsychotic activity of mGlu2/3 agonists could in part rely on their ability to functionally antagonize the colocalized 5-HT_2A_ receptors and, conversely, that pathological alterations of the mGlu2/3–5-HT_2A_ receptor–receptor coupling could account for developing schizophrenia (Marek et al., 2001; González-Maeso et al., 2008; Moreno et al., 2016).

mGlu2/3 autoreceptors and 5-HT_2A_ heteroreceptors also exist in spinal cord glutamatergic nerve endings, at the presynaptic level, where they hetero-dimerize in an antagonist-like fashion to control glutamate exocytosis. Due to the relevance of presynaptic release-regulating autoreceptors in the modulation of synaptic strength at glutamatergic synapses, the mGlu2/3 – 5-HT_2A_ cross talk might represents an innovative target for drugs that modulates the efficiency of fast synaptic transmission in the spinal cord. In particular, we proposed that 5-HT_2A_ antagonists might act as “Indirect Positive Allosteric Modulators” (IPAMs) of the mGlu2/3 receptors, since blockade of the presynaptic release-regulating 5-HT_2A_ receptors would indirectly strengthen the functional outcomes of the colocalized presynaptic release-regulating glutamate receptors (Olivero et al., 2018). To verify the hypothesis Olivero and colleagues (2018) tested the impact of few 5-HT_2A_ antagonists, including trazodone (TZD) on the expression and the functions of mGlu2/3 autoreceptors. TZD was developed as antidepressant and anxiolytic drug (Stahl, 2009), but so far its pharmacological profile is not fully elucidated. The drug inhibits the serotonin transporters and blocks the 5-HT_2_ receptors (the 5-HT_2A_ and 5-HT_2C_ receptor subtypes), but also exerts antagonistic effects against 5-HT_1A_ receptors, α -adrenergic receptors, and H-histaminergic receptors (reviewed by Fagiolini et al., 2012). By blocking 5-HT_2A_ heteroreceptors, “*in vitro*” TZD was found to reinforce the mGlu2/3 inhibitory effects on glutamate exocytosis (Olivero et al., 2018).

Data in the literature suggest that glutamate exocytosis, as well as mGlu2/3 receptor expression and functions are altered in animal suffering from the chronic sciatic ligation (Dubner and Ruda, 1992; Dickenson et al., 1997). The present study aims at investigating whether the expression and the functions of the presynaptic release-regulating mGlu2/3 and 5-HT_2A_ receptors in spinal cord glutamatergic nerve endings (we refer to as synaptosomes) are altered in animal suffering from the chronic sciatic ligation and if oral acute trazodone could impact mGlu2/3 functions recovering the altered glutamate exocytosis.

## Materials and Methods

### Animals

Experiments were performed on male CD^®^IGS rats weighing 190 to 250 g (Charles River, Italy). The animals were housed at Porsolt (Le Genest-Saint-Isle, France) in groups of 5 rats in macrolon cages until surgery with free access to food and water under a 12/12 h light/dark cycle (light cycle: 7:00 am to 7:00 pm.

### Chronic Constriction Injury, Quantification of Tactile Allodynia, and Trazodone Treatment

The surgery was carried out at Porsolt. Under anaesthesia (combination of ketamine and medetomidine), the sciatic nerve of the left hind paw was exposed at the level of the middle of the thigh by dissection through biceps femoris. The nerve was freed of adhering tissue, and four ligatures were loosely tied around it with approximately 1 mm spacing. After recovery, on day 6 post-surgery, rats were subjected to a pre-test using tactile stimulation of both hindpaws to verify the presence of neuropathic pain. Only rats responding on the lesioned paw to force between 0% and 30% of the force inducing withdrawal of the non-lesioned paw were included in the experiments. These animals are indicated throughout the text as injured rats, while the control rats are naïve animals. Tactile allodynia was evaluated using the electronic von Frey test (Bioseb, EVF2). The animals were placed under an inverted acrylicplastic box (18 x 11.5 x 14 cm) on a gridfloor. The tip of an electronic von Frey probe was then applied with increasing force first below the non-lesioned and then below the lesioned hindpaw. The force required to induce paw-withdrawal was automatically recorded. This procedure was carried out three times, and the mean force per paw was calculated.

When indicated, on day 7 post-surgery, injured rats were orally treated with trazodone (TZD) at the dose of 0.3 mg/kg (dispersed in 0.5% methylcellulose in distilled water) and sacrificed 1 h after the treatment. The spinal cords (cerebral level to L2 level) were collected and frozen at −80°C in buffered sucrose (0.32 M, pH 7.4, buffered with TRIS 0.01 M). The frozen tissues were sent to DIFAR, Section of Pharmacology and Toxicology to carry out functional and biochemical studies in isolated synaptosomes (Hardy et al., 1983; Dunkley et al., 1988; Franklin and Taglialatela, 2016).

All experimental procedures were approved by Porsolt’s internal ethical review committee and are in accordance with French Government and NIH guidelines.

### Preparation of Synaptosomes

Purified synaptosomes were isolated from the frozen spinal cords of control (naive), injured (animal suffering from sciatic ligation), vehicle-treated injured, and the TZD-treated injured rats as previously described (Musante et al., 2011). Synaptosomes were then resuspended in a physiological solution with the following composition (mM): NaCl, 140; KCl, 3; MgSO_4_, 1.2; CaCl_2_, 1.2; NaH_2_PO_4_, 1.2; NaHCO_3_, 5; HEPES, 10; glucose, 10; pH 7.4.

### Experiments of Transmitter Release

Synaptosomes were incubated for 15 min at 37°C in a rotary water bath in the presence of [^3^H]D-aspartate ([^3^H]D-Asp, f.c.: 50 nM). Identical portions of the synaptosomal suspensions were layered on microporous filters at the bottom of parallel thermostated chambers in a superfusion system (Raiteri et al., 1974; Summa et al., 2013; Ugo Basile, Gemonio, Varese, Italy).

Synaptosomes were transiently (90 s) exposed, at *t* = 39 min, to high KCl containing medium (Di Prisco et al., 2012) in the absence or in the presence of agonists. Fractions were collected as follow: two 3-min fractions (basal release), one before (*t* = 36–39 min) and one after (*t* = 45–48 min) a 6-min fraction (*t* = 39–45 min; evoked release). Fractions collected and superfused synaptosomes were measured for radioactivity.

The amount of radioactivity released into each superfusate fraction was expressed as percentage of the total radioactivity. The KCl-induced overflow was estimated by subtracting the neurotransmitter content into the first and the third fractions collected (basal release, b1 and b3) from that in the 6-min fraction collected during and after the depolarization pulse (induced release, b2). Within the text, the effect of agonists/antagonists is also expressed as percentage of the KCl-induced overflow of tritium observed in the absence of receptor agonists and antagonists (percent of control).

### Immunoblotting

Rat spinal cord purified synaptosomes were lysed in modified RIPA buffer (10 mM Tris, pH 7.4, 150 mM NaCl, 1 mM EDTA, 0.1% SDS, 1% Triton X-100, protease inhibitors) and quantified for protein content. Samples were boiled for 5 min at 95°C in SDS-PAGE loading buffer and then separated by SDS-7.5% PAGE (20–30 μg/lane) and transferred onto PVDF membranes. Membranes were incubated for 1 h at room temperature in Tris-buffered saline-Tween (t-TBS: 0.02 M Tris, 0.15 M NaCl, and 0.05% Tween 20), containing 5% (w/v) non-fat dried milk and then probed with rabbit anti-mGlu2/3 (1:2000), rabbit anti-5-HT_2A_ (1:500) and rabbit anti-GAPDH (1:10000) antibodies overnight at 4°C. The anti-mGlu2/3 antibody recognizes an aminoacidic sequence of the NH_2_ terminus common to both the mGlu2 and the mGlu3 receptor proteins, while the anti–5-HT2A antibody recognizes the NH_2_ terminus (amino acids 22–41) of the 5-HT_2A_ receptor protein. After extensive washes in t-TBS, membranes were incubated for 1h at room temperature with appropriate horseradish peroxidase-linked secondary antibodies (1:20000). Images were acquired using the Alliance LD6 images capture system (Uvitec, Cambridge, UK) and analysed with UVI-1D software (Uvitec, Cambridge, UK).

### Calculations and Statistical Analysis

Multiple comparisons were performed with analysis of variance (ANOVA) followed Tukey’s multiple-comparisons test; direct comparisons were executed by Student’s t-test. Data were considered significant for P < 0.05 at least.

### Chemicals

[2,3-^3^H]D-Asp (specific activity 11.3 Ci/mmol) was from Perkin Elmer (Boston, MA, USA). LY379268 was purchased from Tocris Bioscience (Bristol, UK). (±)-1-(2,5-Dimethoxy-4-iodophenyl)-2-aminopropane hydrochloride ((±)DOI), trazodone, horseradish peroxidase-conjugated anti-mouse, and anti-rabbit secondary antibodies were from Sigma (Milan, Italy). Luminata Forte Western blotting was purchased from Millipore (Temecula, CA, USA). Rabbit anti-GAPDH antibody was from Abcam (Cambridge, UK), rabbit anti-mGlu2/3 antibody was from Novus Biologicals (Littleton CO, USA), rabbit anti–5-HT_2A_ antibody was from Immunostar (Hudson, WI, USA).

## Results

### Impact of the Sciatic Ligation on the [^3^H]D-Aspartate Exocytosis From Spinal Cord Synaptosomes

**Figure 1:**
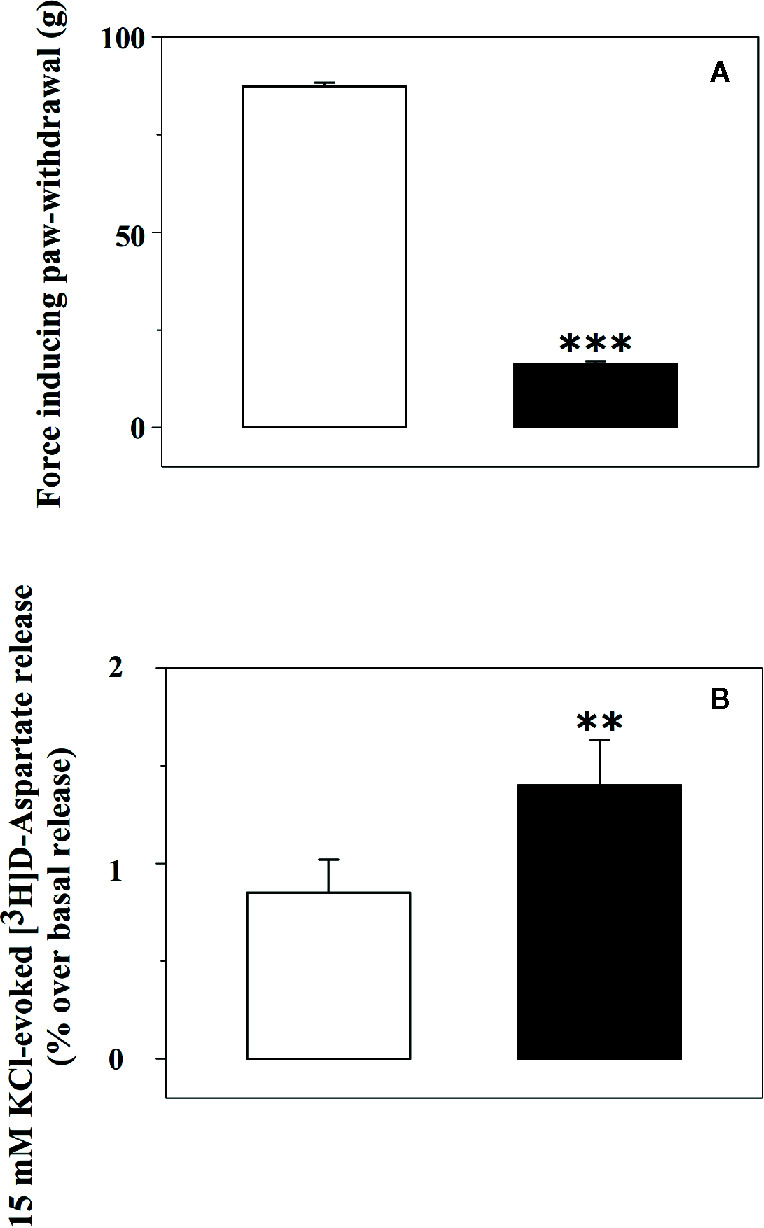
Tactile allodynia correlates with impaired glutamate exocytosis from synaptosomes isolated from the spinal cord of injured rats. **(A)** Effect of chronic constriction injury on tactile allodynia. Control (uninjured, empty bar) and injured (black bar) rats were analysed for Tactile allodynia in the von Frey test. Results represent the tactile thresholds expressed as grams on day 6 following sciatic nerve ligation. Data are mean ± SEM of 32 rats for each group. ***p< 0.001 versus control group (Student’s *t*-test). **(B)** 15 mM KCl-evoked release of [^3^H]D-aspartate from synaptosomes isolated from the spinal cord of control rats and of animals suffering from sciatic ligation. Synaptosomes from control (uninjured, empty bar) and from injured (black bar) rats were label with [^3^H]D-aspartate ([^3^H]D-asp) and exposed in superfusion to 15 mM KCl-enriched medium to trigger exocytosis. The depolarization-evoked release is evaluated as induced overflow, and it represents the amount of tritium released upon the spontaneous release. Results are expressed as mean ± SEM of 8 experiments run in triplicate (three superfusion chambers for each experimental condition). **p< 0.01 versus control (Student’s *t*-test).

The release of glutamate from synaptosomes isolated from the spinal cord of rats suffering from chronic sciatic ligation (injured rats) and of control (uninjured) animals was analysed. Injured animals are those rats that developed on day 6 post-surgery a significant decrease of the paw-withdrawal force in the lesioned side as compared with the non-lesioned paw (−81.0 ± 0.5%, p < 0.001, n = 32 for each group, **Figure 1A**).

Glutamate exocytosis was quantified as 15 mM KCl-evoked overflow of preloaded [^3^H]D-aspartate ([^3^H]D-Asp), a non-metabolizable glutamate analogue routinely used as a marker of the endogenous aminoacid in release studies (Grilli et al., 2004; Di Prisco et al., 2012; Di Prisco et al., 2016). The tritium overflow elicited by the depolarizing stimulus from spinal cord synaptosomes of injured rats was significantly higher than that from spinal cord synaptosomes of control rats (+62.6 ± 9.4, results expressed as percent of increase, p<0.05, n = 6, **Figure 1B**).

### Impact of the Sciatic Ligation on the Presynaptic mGlu2/3 Autoreceptors Controlling [^3^H]D-Aspartate Exocytosis in Spinal Cord Synaptosomes

**Figure 2:**
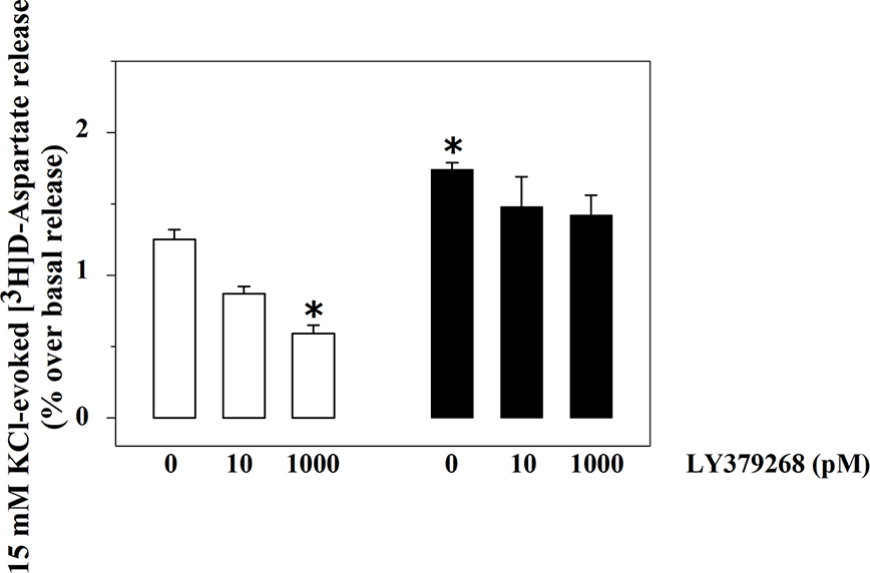
Effects of LY379268 on the 15 mM KCl-evoked release of [^3^H]D-aspartate exocytosis from synaptosomes isolated from the spinal cord of injured rats. Spinal cord synaptosomes were prepared from rats suffering from sciatic ligation (black bar) and from control uninjured (empty bar) rats. Synaptosomes were exposed in superfusion to the 15 mM KCl-enriched medium in the absence or in the presence of LY379268 (concentrations as indicated). Results are expressed as mean ± SEM of 8 experiments run in triplicate. *p< 0.05 versus controls (two-way ANOVA and Tukey’s multiple-comparisons test).

The exocytosis of [^3^H]D-Asp from spinal cord synaptosomes is controlled presynaptically by release-regulating mGlu2/3 autoreceptors (Di Prisco et al., 2016; Olivero et al., 2018). The mGlu2/3 agonist LY379268 (10 and 1000 pM) inhibits in a concentration-dependent fashion the tritium exocytosis in control spinal cord synaptosomes (10 pM, −29.5 ± 6.4, n.s.; 1000 pM, −52.4 ± 7.9, p<0.05, n = 5, results expressed as percent of change). The agonist, however, lost efficacy in controlling glutamate exocytosis in spinal cord synaptosomes from animals suffering from the sciatic ligation (**Figure 2**). Particularly, LY379268 (10 and 1000 pM) failed to affect the 15 mM KCl-evoked [^3^H]D-Asp overflow in these terminals (10 pM, −13.4 ± 6.3 n.s.; 1000 pM, −9.0 ± 12.7, n.s., n = 5, results expressed as percent of change).

### Impact of the Sciatic Ligation on the Presynaptic 5-HT_2A_ Heteroreceptors Controlling [^3^H]D-Aspartate Exocytosis in Spinal Cord Synaptosomes

**Figure 3:**
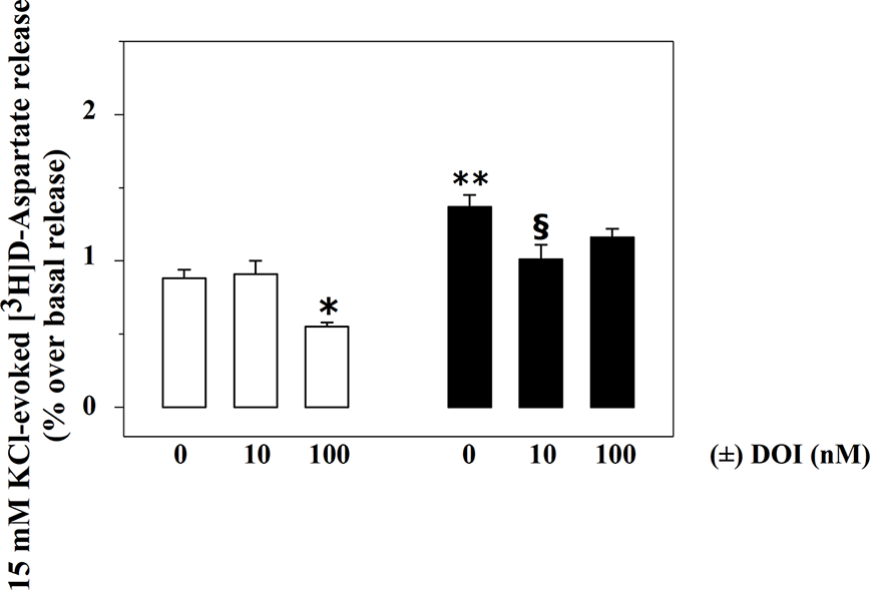
Effects of (±) DOI on 15 mM KCl-evoked release of [^3^H]D-aspartate exocytosis from synaptosomes isolated from the spinal cord of injured rats. Synaptosomes from control (empty bar) and injured (black bar) rats were exposed in superfusion to 15 mM KCl-enriched medium in the absence or in the presence of (±) DOI (concentrations as indicated) as previously described. Results are expressed as mean ± SEM of 5 experiments run in triplicate. *p< 0.05 versus control; **p< 0.01 versus control; ^§^p< 0.05 versus injured rats (two-way ANOVA and Tukey’s multiple-comparisons test).

Rat spinal cord synaptosomes also possess inhibitory, presynaptic, release-regulating 5-HT_2A_ heteroreceptors controlling glutamate exocytosis (Olivero et al., 2018). **Figure 3** shows that 100 nM (±) DOI, a selective 5-HT_2A_ agonist, significantly reduces the 15 mM KCl-evoked release of [^3^H]D-Asp in spinal cord synaptosomes from control rats (−37.5 ± 5.2, p<0.05, n = 6, results expressed as percent of change), being inactive when added at a lower (10 nM) concentration (+ 3.4 ± 5.9, n.s., n = 6, results expressed as percent of change).

Differently, 100 nM (±) DOI slightly, although not significantly, affected the 15 mM KCl-evoked release of [^3^H]D-Asp from the spinal cord synaptosomes of injured rats (−14.6 ± 5.7, n.s., n = 6, results expressed as percent of change). The agonist, however, significantly inhibited tritium overflow when added at 10 nM (−27.9 ± 4.1, p<0.05, n = 6, results expressed as percent of change).

### Impact of Trazodone Treatment on the mGlu2/3 Autoreceptors and 5-HT_2A_ Heteroreceptors in Spinal Cord Synaptosomes From Animals Suffering From Sciatic Ligation

In healthy condition, in spinal cord nerve endings, presynaptic release-regulating 5-HT_2A_ heteroreceptors couple in an antagonist-like fashion the release-regulating presynaptic mGlu2/3 autoreceptors (Olivero et al., 2018). We asked whether blockade of the 5-HT_2A_ heteroreceptors could recover the reduced efficiency of spinal mGlu2/3 autoreceptors in injured rats. As far as the 5-HT_2A_ antagonists are concerned, we focussed on the orally active 5-HT_2A_ antagonist TZD, due to the wide literature describing its pharmacodynamic and pharmacokinetic profile (Cheng et al., 1999; Luparini et al., 2004; Ghanbari et al., 2010).

**Figure 4:**
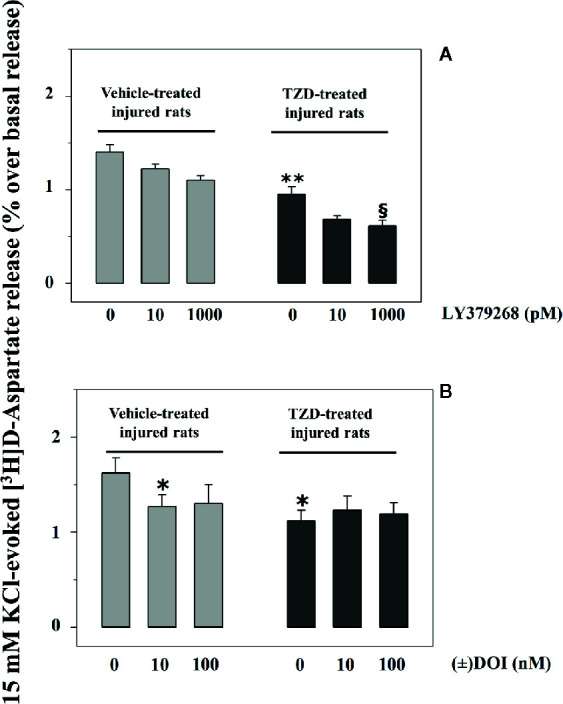
Acute trazodone modifies functionally the presynaptic release-regulating mGlu2/3 and 5-HT_2A_ receptors in synaptosomes isolated from the spinal cord of injured rats. Synaptosomes from TZD-untreated injured animals (vehicle-treated, light grey bar) and from TZD-treated injured rats (dark grey bar) were exposed in superfusion to the 15 mM KCl-enriched medium in the absence or in the presence of LY379268 (A) or of (±) DOI (B, concentrations as indicated). Results are expressed as mean ± SEM of 6 **(A)** and 5 **(B)** experiments respectively run in triplicate. *p< 0.05 versus vehicle-treated synaptosomes; **p< 0.01 versus vehicle-treated synaptosomes; ^§^p< 0.05 versus TZD-treated rats synaptosomes (two-way ANOVA and Tukey’s multiple-comparisons test).

Injured rats were randomly subdivided into two groups, one orally administered TZD (0.3 mg/kg, TZD-treated injured rats) and the other one vehicle (0.5% methylcellulose, vehicle-treated injured rats). The glutamate exocytosis from spinal cord synaptosomes isolated from TZD-treated injured rats was significantly lower than that from vehicle-treated injured rats (**Figures 4A, B**). Vehicle administration did not modify on its own the 15 mM KCl-evoked release of tritium from control rats (not shown).

LY379268 (10–1,000 pM) failed to affect significantly the 15 mM KCl-evoked release of [^3^H]D-Asp in spinal cord synaptosomes from vehicle-treated injured rats (compare **Figure 2** and **Figure 4A**). Differently, 1000 pM LY379268 significantly inhibited the [^3^H]D-Asp exocytosis from spinal cord synaptosomes isolated from TZD-treated injured rats, while 10 pM LY379268 slightly, although not significantly, reduced it (**Figure 4A**).

Experiments were also dedicated to test the impact of (±) DOI on glutamate exocytosis from TZD-treated spinal cord synaptosomes. The oral TZD administration reduced the 15 mM KCl-evoked release of tritium from spinal cord synaptosomes from injured rats when compared to vehicle-treated animals. The glutamate exocytosis from these terminals, however, was not affected by the 5-HT_2A_ agonist (**Figure 4B**).

### Impact of the Chronic Sciatic Ligation on the Expression of mGlu2/3 and 5-HT_2A_ Receptor Proteins in Spinal Cord Synaptosomes

**Figure 5:**
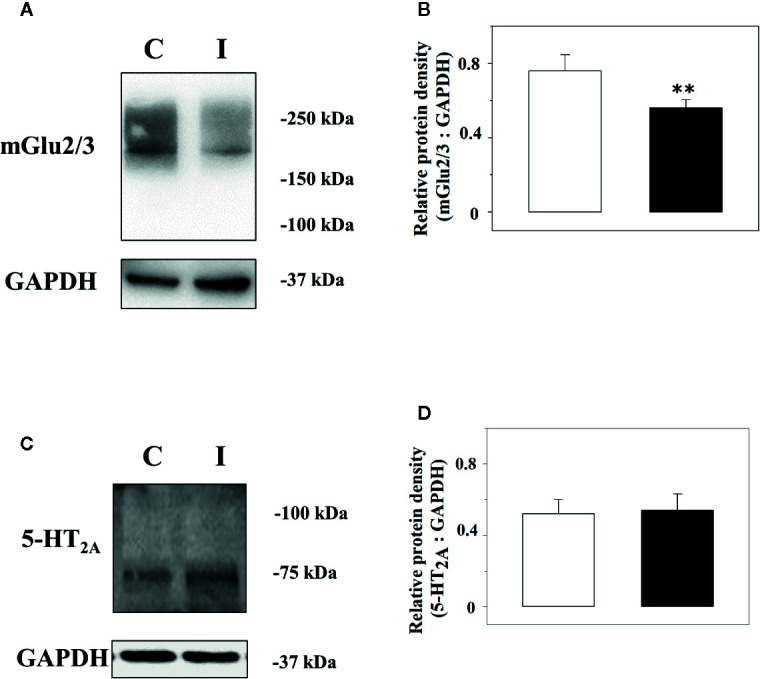
Western Blot analysis of the mGlu2/3 and the 5-HT_2A_ receptor proteins in synaptosomal lysates from the spinal cord of control and injured rats. Left panels: Representative western blot showing modifications in mGlu2/3 **(A)** and of the 5-HT_2A_ **(C)** receptor proteins in spinal cord synaptosomal lysates from rats suffering from sciatic ligation (injured, I) and from control uninjured (control, **C**) rats. The blot is representative of five (mGlu2/3) and 7 (5-HT_2A_) analyses carried out in different days. Right panels: quantification of the mGlu2/3 ÷ GAPDH ratio **(B)** and of the 5-HT_2A_ ÷ GAPDH ratio **(D)** in synaptosomes isolated from controls (white bar) and injured (black bar) animals. Results are expressed as mean ± SEM. **p<0.01 vs control, (Student’s *t*-test).

Immunochemical studies were carried out to quantify the expression of the mGlu2/3 and the 5-HT_2A_ receptor proteins in the lysates of spinal cord synaptosomes from injured rats. The anti-mGlu2/3 antibody recognized a band of ∼220 kDa in both the spinal cord synaptosomal lysates from control and injured rats, but not at ∼100 kDa that represents the expected weight of the receptor subunit, consistent with the existence of the mGlu2/3 dimeric form of the receptor in these terminals (**Figure 5A**). Differently, the anti–5-HT_2A_ antibody unveiled a component in the synaptosomal lysate with a mass (∼ 75 kDa) corresponding to the monomeric form of the receptor (**Figure 5C**). GAPDH was used as an internal control (**Figures 5A, C**). The receptor protein signals were expressed as mGlu2/3 ÷ GAPDH or 5-HT_2A_ ÷ GAPDH ratio (**Figures 5B, D**). The results showed a significant reduction of the mGlu2/3 ÷ GAPDH ratio value in injured animals (−27.6 ± 5.6%, results expressed as percent of change, p < 0.05; n = 5, **Figure 5B**) in 5 lysates out of the 9 synaptosomal preparations analyzed (**Figure 5B**) when compared to control, while an increase (+30.3 ± 9.8, results expressed as percent of change, p< 0.05, not shown) was observed in 3 lysates out of the 9 and no change (+ 4.2, result expressed as percent of change, not shown) in 1 synaptosomal lysate. Differently, the 5-HT_2A_ ÷ GAPDH ratio value in injured animals was comparable to that in control rats in all the preparations analyzed (**Figure 5D**).

### Impact of Trazodone Treatment on the mGlu2/3 Receptor Proteins in Spinal Cord Synaptosomes From Injured Rats

**Figure 6:**
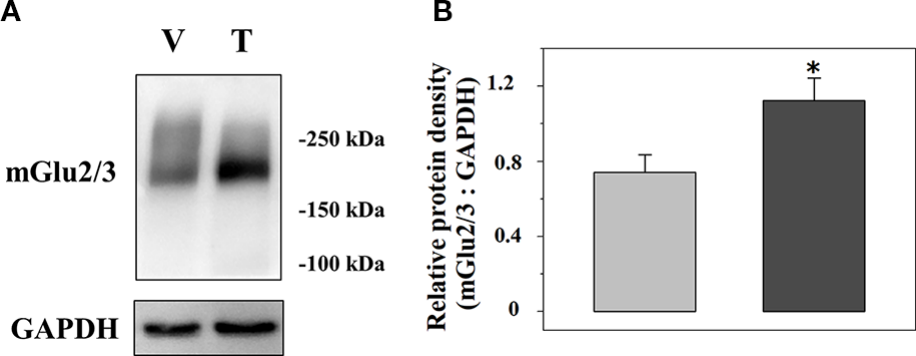
Acute trazodone modifies the expression of the presynaptic release-regulating mGlu2/3 autoreceptors in synaptosomes isolated from the spinal cord of injured rats. Western Blot analysis **(A)** and quantification **(B)** of the mGlu2/3 receptor proteins expression in synaptosomal lysates from TZD-treated injured rats (T) when compared to vehicle-treated injured rats (V). The GAPDH protein was used as an internal control. The blot is representative of five blots. **(B)** TZD-treated injured animals (dark grey bar) and vehicle-treated injured rats (light grey bar). Results are expressed as the mean of the mGlu2/3 ÷ GAPDH ratio. *p<0.05 vs control, (Student’s *t*-test).

We analysed the expression of mGlu2/3 receptor proteins in TZD-treated injured spinal cord synaptosomal lysates when compared to vehicle-treated injured animals. A representative blot is reported in **Figure 6A** and the mean values of the mGlu2/3 ÷ GAPDH ratio for both the vehicle-treated and the TZD-treated injured rats are reported in **Figure 6B**. The results from 10 lysates out of 12 showed a significant increase of the mGlu2/3 ÷ GAPDH ratio when compared to controls (+48.8 ± 10.1, p< 0.05; results expressed as percent of change).

## Discussion

Spinal cord hyper-glutamatergicity contributes to the development of central hyperalgesia in animals suffering from the chronic constriction of the sciatic nerve. The altered glutamatergic transmission might depend on several concomitant maladaptive events, including changes in the efficiency of transmitter exocytosis from nerve terminals (and astrocytes/glial cells as well) and altered expression/functions of the presynaptic release-regulating autoreceptors.

As far as the glutamate release efficiency is concerned, its dysregulation was suggested by evidence in the literature demonstrating impaired glutamate content and exocytosis from isolated nerve endings of the spinal cord of lesioned animals (Somers and Clemente, 2002; Morioka et al., 2015; Wang et al., 2015) and it is here confirmed by the results showing increased glutamate exocytosis from the synaptosomes isolated from the spinal cord of rats suffering from the sciatic ligation.

In this context, checking the efficiency of presynaptic inhibitory glutamatergic release-regulating autoreceptors became mandatory when considering that the loss of efficiency of these receptors could further worsen the pathological framework.

Among glutamate receptors, the mGlu belonging to the group II function as inhibitory release-regulating autoreceptors in the CNS of mammals, including the spinal cord (Gerber et al., 2000; Di Prisco et al., 2012; Di Prisco et al., 2016). These receptors preferentially locate nearby the site of transmitter exocytosis and are activated by glutamate exceeding the physiological level and diffusing beyond the synaptic active zone (Olivero et al., 2019; Pittaluga, 2019).

A large body of evidence published in the last two decades supports the main role of mGlu2/3 receptors as regulators of glutamate release from primary afferent fibres in the dorsal horn of the spinal cord (Gerber et al., 2000) as well as main players in analgesia (Simmons et al., 2002; Chiechio et al., 2009; Chiechio et al., 2010; Bernabucci et al., 2012; Chiechio and Nicoletti, 2012). The data so far available concerning their expression in the spinal cord of animals suffering from neuropathic pain, however, are conflicting. A significant reduction of the mGlu2/3 receptor expression was detected in and around the lesion site in spinal cord injured rats, although it was uncertain in which cell types the changes occurred, i.e. neuronal or glial ones (Mills et al., 2001). Differently, significant changes in the expression of mGlu2/3 receptor proteins in the lumbar segment of the spinal cord of animals suffering from the monolateral chronic sciatic constriction did not emerge soon after the induction but several (3 to 7) days after the lesion (Chiechio et al., 2002).

Based on the assumption that the increased glutamate release could depend on the reduced efficiency of the presynaptic autoreceptors in controlling the vesicular exocytosis, we analysed the efficiency of LY379268 in inhibiting glutamate overflow from spinal cord synaptosomes of lesioned rats 7 days after the sciatic ligation. In line with the hypothesis, our functional results showed a significant loss of efficacy of the agonist in controlling glutamate exocytosis that in a large percentage of the animals was paralleled by a reduced insertion of the receptor protein in synaptosomal plasmamembranes.

Besides genetic and epigenetic mechanisms of control of the mGlu2/3 receptors expression (Chiechio et al., 2009), we recently demonstrated that their insertion in synaptosomal plasmamembranes as well as their releasing activity is controlled by mechanisms of “metamodulation” (Olivero et al., 2019). The term “metamodulation” refers to the mechanism(s) of control of synaptic plasticity based on the functional crosstalk linking two receptors colocalized on the same nerve endings (Sebastião and Ribeiro, 2015). As far as the glutamatergic spinal cord synaptosomes are concerned, we demonstrated that the mGlu2/3 autoreceptors colocalize and functionally cross-talk in glutamatergic nerve endings with the 5-HT_2A_ heteroreceptors. Particularly, the receptor-receptor interaction assures the serotonergic-induced, antagonist-like regulation of the mGlu2/3 receptors controlling glutamate exocytosis (Olivero et al., 2018).

The role of serotonin and 5-HT_2A_ receptors in controlling pain perception has been matter of study and the involvement of the descending serotonergic pathway and of the 5-HT_2A_ receptors in the spinal cord sensitization is supported by data in the literature. In 1980, Proudfit demonstrated that lesions of the raphe magnus resulted in decreased nociceptive thresholds and attenuation of morphine-induced analgesia (Proudfit, 1980). In 2007, Okamoto and colleagues highlighted the role of 5-HT_2A_ receptors in the control of nociceptive neural activities (Okamoto et al., 2007). More recently, Liu and colleagues (2010) proposed that changes in the descending inhibitory 5-HT system occur upon spinal nerve injury and participates to central sensitization and pain perception. Furthermore, Lopez-Alvarez and colleagues (2018) reported the altered expression of the 5-HT_2A_ receptors in the spinal cord dorsal horn following sciatic nerve transection in rats 2 weeks after injury. Finally, data exists in the literature describing the efficacy of the 5-HT_2A_ antagonist TZD to attenuating pain perception in rats suffering from chronic constriction (Okuda et al., 2003) and in humans suffering from diabetic neuropathy (Wilson, 1999).

An interesting result of the present study is that, opposite to LY379268, the 5-HT_2A_ agonist (±) DOI does not lose efficacy in controlling presynaptically glutamate exocytosis in spinal cord synaptosomes from injured rats. Rather, in these rats, its potency is “apparently” increased, since a concentration one-fold lower than that effective in healthy conditions caused significant inhibition of glutamate outflow. Since evident changes in the 5-HT_2A_ receptors protein expression in the synaptosomal lysates did not emerge, we speculated that the functional adaptation of the presynaptic serotonergic heteroreceptors could be explained by assuming that the 5-HT_2A_-mGlu2/3 balance was impaired in the spinal cord of injured rat, and, particularly, that the serotonergic tone became predominant in the receptor-receptor cross-talk, silencing the colocalized mGlu2/3 autoreceptors. The hypothesis is supported by the release studies that unveiled a loss of function of the presynaptic mGlu2/3 autoreceptors concomitant to the gain of function of the 5-HT_2A_ heteroreceptors.

Well in line with the hypothesis, we found that the acute oral administration of TZD, i.e. a treatment that would allow a systemic blockade of the 5-HT_2A_ receptors, including those located in the spinal cord glutamatergic nerve endings, recovered almost all the molecular impairments observed in injured rats. In particular, the acute oral administration of TZD i) reduced the glutamate exocytosis from spinal cord terminals, ii) silenced the presynaptic release-regulating 5-HT_2A_ heteroreceptors, iii) recovered the efficiency of the presynaptic mGlu2/3 autoreceptors. All these events could be tentatively explained by assuming that the “*in vivo*” antagonism of the 5-HT_2A_ heteroreceptors was “memorized and retained” by glutamatergic nerve endings, emerging in “ex vivo, *in vitro*” release studies as loss of function of the presynaptic 5-HT_2A_ heteroreceptors and concomitant gain of function of the coupled mGlu2/3 release-regulating autoreceptors (see for a recent review Pittaluga, 2019).

An intriguing observation is that the mGlu2/3 receptor proteins in spinal cord synaptosomal lysates from TZD-treated injured rats were significantly increased when compared to control. In an attempt to find a rationale for the changes in the receptor protein content, one might hypothesize that the acute (1 h) oral administration of a low dose (0.3 mg/kg) of TZD could have modified the expression of the mGlu2/3 receptor proteins in spinal cord glutamatergic nerve endings. An alternative hypothesis, however, considers the possibility that the blockade of the 5-HT_2A_ counterpart within the intra-group heteromeric complex could stabilize mGlu2/3 receptors in plasmamembranes, slowing their internalization and degradation in nerve terminals. Despite the timing of the TZD treatment seems best in line with the second hypothesis, the data so far available are insufficient to propose a mechanism accounting for this effect.

To conclude, the findings described in this study confirm that mGlu2/3 autoreceptors in spinal cord glutamatergic nerve endings of rats suffering from the sciatic ligation undergo selective functional adaptations that minimize their role as modulators of glutamate transmission. Furthermore, they demonstrate that the early spinal glutamatergic maladaptation in lesioned animals can be recovered by administering acutely 5-HT_2A_ antagonists. Interestingly, the analgesic effect is observed following the administration of a dose (0.3 mg/kg) of TZD that is two order of magnitude lower than those (20 to 80 mg/kg) found to ameliorate the thermal hyperalgesia in rats suffering from sciatic ligation (Okuda et al., 2003). The apparent discrepancy might rely on differences in the drug administration protocols (TZD was administered at day 7 post injury in the present study and at day 15 post injury in the study of Okuda and colleagues) as well as in the test applied to quantify pain (tactile vs thermal allodynia). Further investigations are needed to correctly address this point.

It is proposed that responses to nociceptive stimuli can be transformed into memories if they cause long-lasting, activity-dependent changes in synaptic strength. Our findings suggest that, among the molecular events accounting for the early maladaptation, impaired mGlu2/3 – 5-HT_2A_ metamodulation could be relevant to the sensitization of nociceptive dorsal horn neurons. Drugs that could restore the pathological unbalance between the two receptors can recover the physiological neuromodulation of the spinal glutamate transmission, reducing pain perception.

Reinforcing the functioning of the mGlu2/3 receptors is recognized as a successful approach to treat the development of pain and, accordingly, different classes of therapeutics have been proposed to this aim, including substances that modulate epigenetically the expression of the group II receptor protein (Chiechio et al., 2002; Chiechio et al., 2009; Chiechio et al., 2010; Bernabucci et al., 2012; Chiechio and Nicoletti, 2012; Zammataro et al., 2014). Although further studies are needed to definitively prove the efficacy of the 5-HT_2A_ antagonists for the cure of spinal pain, our results support their use as alternative therapeutic approach to modulate the mGlu2/3-mediated signalling in pathological conditions associated to neuropathic pain.

## Data Availability Statement

All datasets presented in this study are included in the article/supplementary material.

## Ethics Statement

The experimental procedures in animals were reviewed and approved by Porsolt’s internal ethical review committee and are in accordance with French Government and NIH guidelines.

## Author Contributions

AP designed the experiments, supervised the execution of the research activity and the statistical analysis, and wrote the manuscript. FC, AR, and GO performed release experiments and western blot analysis. BG, ST, and FG made the tissues available, supported the scientific data analysis and discussion, and revised the manuscript. FC, AR, GO, BG, ST, and FG approved the final version of the manuscript and agree to be accountable for all the aspects of the work.

## Funding

The study was granted by Angelini S.p.A. [contract. 039(1)PD18114].

## Conflict of Interest

BG, ST, and FG were employed by the company Angelini Pharma S.p.A.

The remaining authors declare that the research was conducted in the absence of any commercial or financial relationships that could be construed as a potential conflict of interest.

The authors declare that this study received funding from Angelini S.p.A. The funders had the following involvement with the study: they made the tissues available, participated to the data analysis, decision to publish, preparation of the manuscript, discussed and revised the manuscript.

## Abbreviations

[3H]D-Asp, [3H]D-aspartate; CNS, central nervous system; IPAMs, Indirect Positive Allosteric Modulators; mGlu2/3 receptor, metabotropic glutamate receptor type 2/3; 5-HT2A, serotonin type 2A; TZD, trazodone.
